# Long-term dynamics of mind wandering: ultradian rhythms in thought generation

**DOI:** 10.1093/nc/niz007

**Published:** 2019-06-08

**Authors:** Chie Nakatani, Benjamin Ganschow, Cees van Leeuwen

**Affiliations:** 1 Brain and Cognition Research Unit, KU Leuven, Tiensestraat 102 - Box 3711, Leuven, Belgium; 2 Center for Cognitive Science, TU Kaiserslautern, Erwin–Schrödinger-Straβe 52, Kaiserslautern, Germany

**Keywords:** spontaneous thought, experience sampling, cognitive control

## Abstract

Using the method of experience sampling, we studied the fluctuations in thought generation and cognitive control strength during the wakeful hours of the day, centered around episodes of mind wandering. Thought generation, measured in terms of the number of thoughts that concurrently occupy the mind at sampling time, goes through regular 4–6 h cycles, suggesting the mind operates with an alternation of focused and multitasking modes. Cognitive control strength rises and falls in relative coordination with thought generation, implying that both are occasionally misaligned. This happens, in particular, when cognitive control suddenly drops after having been keeping up with a cycle of thought generation. When this drop occurs while the thought generation cycle is still up, mind wandering appears. As cognitive control quickly resumes before returning to intermediate values, the thought generation cycle begins to fall again, and the mind wandering episode comes to an end. Implications regarding the role of long-term regulation in mind-wandering processes are discussed.

## Introduction

Since Thomas Hobbes coined the term “train of thought,” spontaneous mental activity is commonly understood as a sequential process, in which one idea leads to another. Often, however, we have more than one thought on our mind. Most likely, our thought generation rate varies over time and we dwell on certain thoughts longer than on others. The net effect is that the number of thoughts that occupy our mind varies. We are aware of this, when at times we casually say things like: “I have so many things to think about” or, at others: “I was so much focused on that single thought.” While people are known to express such first-person, metacognitive reports spontaneously, they could also be prompted to do so, for instance by asking them to report: “how many things are on your mind right now?” Doing so repeatedly with certain intervals may give us useful information about systematic fluctuations in the thought process.

Besides in number, there are also fluctuations in the extent the thoughts on our mind cohere. Sometimes, thoughts are centered on a single problem; sometimes our mind wanders around topics seemingly unrelated to each other. Cast in terms of loss of sustained attention to a task, the itinerant mind has extensively been studied in the vigilance literature (e.g. Mackworth 1964; Davies and Parasuraman 1982; Oken *et al.* 2006; Thomson *et al.* 2015; Bermudez *et al.* 2016). In this literature, mind wandering starts unintentionally. As the task requires sustained focus, effort is made to bring the task-irrelevant thought down and refocus on the task (Smallwood and Schooler 2006). This brings an end to, what the authors call, a mind-wandering cycle.

Mind wandering is not necessarily destructive; it shares with creative processes the feature of unchecked productive thinking (Ellamil *et al.* 2012). Positive effects may help explain, at least, in part, why our mind wanders rather frequently. The incidence of mind wandering in experimental settings varies from approximately 15% of the time during verbal fluency and memory encoding tasks (Smallwood *et al.* 2003), up to nearly 50% of the time during a simple signal detection task (Antrobus 1968; Giambra 1995; Smallwood *et al.* 2004), i.e. mind wandering is roughly inversely proportionally to task load. Outside of the laboratory, reported incidence rates of mind wandering may occupy 30–40% of thought processes in daily life (Klinger and Cox 1987). These incidence rates suggest that might wandering might have some restorative or homeostatic function, such as in maintaining the integrity of our self-concept (Smallwood and Andrews-Hanna 2013).

Spontaneous (self-generated) and unconstrained (stimulus or task-independent) thought also plays a prominent role in daydreaming (Singer 1966; Antrobus 1968; Klinger and Cox 1987; Klinger 2009; Ellamil *et al.* 2012; Perkins *et al.* 2015). We may consider mind wandering and daydreaming as belonging to a continuous spectrum of spontaneous thought processes (Voss and Voss 2014; Christoff *et al.* 2016). This spectrum, according to Christoff *et al.* (2016) consists of two dimensions. One is strength of “deliberate constraints” implemented though cognitive control. A variety of thought processes can be ordered along this dimension, successively: dreaming, mind wandering, creative thinking, and goal-directed thought. The mind wandering cycle as envisaged by Smallwood and Schooler (2006)—being focused on a task, drifting away from the focus, followed by reflexive awareness of mind wandering and resumption of focused thought—can be described along this dimension as temporary relaxation of deliberate constraints.

The other dimension is strength of “automatic constraints” which involves mechanisms such as saliency that operate outside of cognitive control. In particular, the number of thoughts changes along this dimension. When these constraints are strong, only a small number of distinct thoughts will be salient enough to occupy the mind. In the extreme case, the mind will be in a state of rumination or obsessive thought. As the constraints relax, the number of distinct thought increases, allowing creative thinking, mind wandering, or day dreaming to arise. We will refrain from using the term “automatic,” and adopt “number of thoughts” as our preferred label for this second dimension.

### Trajectories of spontaneous thought processes

**Figure 1.**
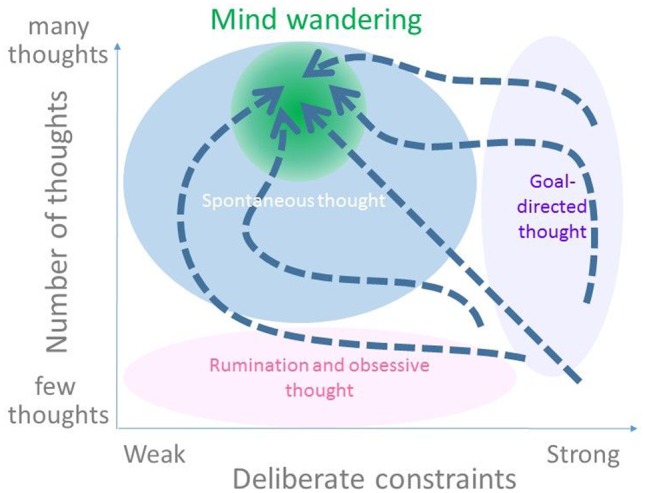
Illustration of different evolutions of the mental state leading to mind wandering, as depicted in a putative 2D state space, adopted and modified from Fig. 1 in Christoff *et al.* (2016). The horizontal axis shows the level of cognitive control, corresponding to Christoff *et al.*’s (2016) notion of strength of deliberate constraints. The vertical axis shows the number of thoughts in mind, corresponding to their notion of strength of automatic constraints. The mind-wandering region (shaded in green) falls within the specter of spontaneous thought. Different trajectories (represented as dotted arrows) lead toward the mind-wandering region.

The two dimensions as considered by Christoff *et al.* (2016) constitute a space in which a point, depending on its location with respect to the axes, may specify an individual’s state of mind at a certain time. Changes in mental state over time could be described as trajectories in this space (Fig. 1). Various trajectories could, in principle, lead to mind wandering; they are not necessarily confined to a fixed pattern. For instance, a specific task in daily life can set off mind wandering if the number of thoughts increases while the focus on the task decreases. This implies a more or less straight trajectory from the lower-right region of the space of Fig. 1 to the upper-left, where fully evolved mind-wandering states occur. Alternatively, the mind-wandering state may be reached, for instance, from the upper-right region of the space, where the control is applied over many thoughts, e.g. in multitasking; or even, in principle, via the lower-left region, where both the control and number of thoughts are low, i.e. rumination or obsessive thinking. Possible trajectories leading to mind wandering vary to the extent in which reduction of control leads or lags the increase in number of thoughts.

Observing trajectories like in Fig. 1 will allow us to raise simple yet unanswered questions about the time course, intra- and inter-individual consistency and variation of the processes leading to mind wandering. To observe these trajectories, the number of thoughts and the level of deliberate constraints need to be measured *repeatedly over time*. This could be done, in principle, in various temporal resolutions and time scales. In the laboratory, mind-wandering-related processes have frequently been studied using magneto– or electro–encephalogram (MEG or EEG) of which time scale is in the range of milliseconds, and using behavioral responses and functional magnetic resonance imaging (fMRI) of which time scale is in the range of seconds (Baird *et al.* 2014; Hellyer *et al.* 2014; Ibáñez-Molina and Iglesias-Parro 2014; Christoff *et al.* 2016; Andrews-Hanna *et al.* 2018). Mind-wandering processes on the scale of minutes and hours have been studied using online questionnaire sessions (Kane *et al.* 2007, 2017). Kane *et al.* (2017) sent questionnaires to participants via a mobile digital device eight times/day for 7 days, to sample the probability and context of mind wandering. The results were correlated with laboratory cognitive measures of executive functions and personality traits. Unfortunately, their study did not report fluctuations in mind-wandering frequency across the sampling period. No measure was taken of numbers of thoughts, preventing the reconstruction of mind-wandering trajectories in the requisite 2D space. We aim to reconstruct the typical trajectory of the mind-wandering process, based on time series of subjective reports. We collected these reports using an experience sampling method, by sending probes to participants’ smartphones during the wakeful hours of the day. Accordingly, the time scale for the trajectory is in the order of hours.

## Methods

### Participants

A total of 32 participants from the psychology program at KU Leuven (69% female, mean age = 19) participated to the study for course credits. None of them reported existing physical and mental health issues.

### Research ethics

The Research Ethics Committee of the Faculty of Psychology and Educational Sciences at KU Leuven approved the current study. The study was preregistered on the Open Science Framework (OSF) as “The Mind Wandering Project” on 30 January 2017 (https://osf.io/vruut/? view_only=07156c32873a4318bf90ec4b9d1c15ab). Data are stored in the OSF site and available on request.

### Probe design

Participants received a short message on their own smartphone. The message included a link to a probe. The probe consisted of a visual task and a questionnaire. Both the task and questionnaire were emulated on participants’ own smartphone. The task involved rating of scene closeness which, as explained below, is used to estimate strength of deliberate constraints. The questionnaire asked participants to briefly report the number and the type of content of their thoughts.

#### Scene closeness rating

To be suitable for experience sampling, a test of cognitive control needs to be simple and one-shot. This means, we cannot use standard tests like the Sustained Attention to Response Task (SART). Such a task would have required key presses to “go” stimuli, e.g. a digit “3,” as opposed to other digits. “Go” responses to “no-go” stimuli in this task indicate failure of sustained task-related attention (Robertson *et al.* 1997; Manly *et al.* 1999). To obtain a number of error trials sufficient for evaluating sustained attention, the task has to be continued for at least 8 min (Nuechterlein *et al.* 1983; Temple *et al.* 2000). This renders it unsuitable for our purpose.

**Figure 2.**
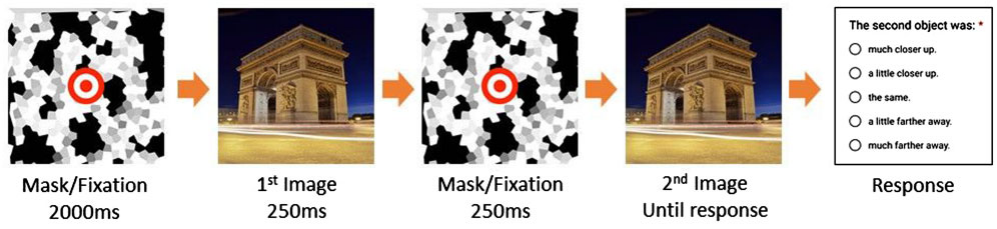
Scene closeness rating task. The mask/fixation display is presented for 2000 ms, followed by a 250 ms presentation of a scene, again the mask/fixation for 250 ms, then the same scene as is shown again until the participant swipes down the phone display. The participant is asked to judge whether the object is closer/farther away the second time than the first time.

We therefore developed a single-trial test of cognitive control. In this test, the same scene is presented on a smartphone display twice, both times exactly the same size (Fig. 2). Participants are not informed of the size identity, and are asked to report how much closer/farther the second presentation appeared relative to the first one, using a five-point scale. Despite the identity, participants are expected not always to give “same” responses, i.e. the midpoint of the five-point scale (approximately 25% non-same responses were given in the current study and a follow-up in Supplementary materials, Part 1). Non-same responses are taken to indicate strong cognitive control, imposing a top-down bias to overrule the default “same” response. Nondefault responses were therefore taken to reflect deliberate constraints.

Stimuli were realistic color images of scenes, collected from the public domain image repository (https://pixabay.com). Seventy-one scene images with one main object, e.g. a red robin on a tree branch, were chosen for 71 times of probing. The images were selected by two of the authors independently (B.G., male and C.N., female) for good visibility under various viewing conditions during real-life sampling and for similar levels of salience. Images approved by both judges were selected for inclusion. The images were formatted to 700 × 700 pixels and collated to GIF animation. The sequence of events is illustrated in Fig. 2. On the smartphone display, a fixation/mask was presented for 2000 ms, followed by a scene of a single object and background which is displayed for 250 ms. The fixation display masks the image for 250 ms. Then the scene was presented for the second time. Presentation timing error was 1.4 ms for 250 ms in a benchmark test using two types of smart phones which are popular to the local community.

The scene closeness rating task was given at the beginning of the probe. Participants were instructed not to change viewing distance during the test and to judge whether the second time, the object is “much farther away,” “a little farther away,” “the same,” “a little closer up,” or “much closer up,” compared with the first presentation. After the response, participants were asked to report any problems that may have occurred to the stimulus presentation (e.g. noticeable slowing down of image display rate or a missing second image).

Although the task closely resembles the Boundary Extension test (Mullally *et al.* 2012; Mullally and Maguire 2014), its focus and use of data differs quite markedly from the current one. An analysis centered on non-same closeness ratings is applied for the first time in the current study. Therefore, we assessed its reliability in a separate study using 20 different participants, in an effort to replicate the main results. Details are reported in Supplementary materials, Part 1: A reliability study for scene closeness rating test.

#### Questionnaire

*Number of thoughts in mind.* The scene closeness rating task is followed immediately by the questionnaire. The first question asks to report the number of thoughts the participant had in mind just before arrival of the probe. Participants were instructed to report the number of topics rather than individual content items. This was illustrated with examples as follows: when thinking of your lunch while attending a lecture, the report should be one (lunch) or two (lunch and lecture), rather than five (sandwich, apple, cookies, friends, and lecture slides). Our volunteers were able to report such numbers without confusion. The number is reported by selecting the response alternative from a dropdown menu, running from 0, 1, 2, 3, 4, 5, 6, 7, 8, 9, to 10+ (Table 1, Question 1).

**Table 1:** Questionnaire items

Questions	Response type
1. How many things were on your mind?	Dropdown from 0, 1, 2, 3, 4, 5, 6, 7, 8, 9, 10+
2. Was your mind focused on thoughts relevant to what you were doing?	Multiple choice A. Yes, I was focused. B. My mind was wandering to thoughts not relevant to what I wanted to do. C. I didn’t need to be focused on anything specific and my mind drifted.
3. How many minutes did you experience mind wandering?	Alphanumeric
4. How clear or vivid was your mind-wandering experience?	Five-point scale between ‘Very unclear’ and ‘Very clear’
5. How enjoyable were your thoughts?	Five-point scale between ‘Not at all enjoyable’ and ‘Very enjoyable’
6. How likely could these thoughts actually happen in your life?	Five-point scale between ‘Not at all likely’ and ‘Very likely’
7. Were you thinking about the past, the present or the future?	Five-point scale between ‘Far into the past’ and ‘Far into the future’
8. How often do you have these particular thoughts?	Five-point scale between ‘Not at all often’ and ‘Very often’
9. How occupied or full was your mind with these thoughts?	Five-point scale between ‘Not at all occupied’ and ‘Completely occupied’
10. To what main activity did you mind wander to?	Free text
11. Whom was your mind wandering about?	Multiple choice A. Yourself B. Family member(s) C. Partner (girlfriend/boyfriend) D. Friend(s) E. Acquaintance(s) F. Nobody in particular G. Other: Free text
12. When the phone rang, I was trying to focus on something …	Multiple choice A. Work related B. School related C. Social (parties, meeting friends, planning trips, family visits, etc.) D. Commuting E. Nothing special F. Other: free text
13. How tired did you feel when you were mind-wandering?	Five-point scale between ‘Very tired’ and ‘Very awake’
14. How were you feeling when you were mind-wandering?	5-point scale between ‘Very unhappy’ and ‘Very happy’
15. Are you under the influence of alcohol or other mind-altering substances?	A. Yes B. No

*State of mind and qualitative aspects of mind wandering.* In the rest of the questionnaire, participants reported their mental state. First, they report if just before the arrival of the probe they were focused or mind wandering. In case of mind wandering, they also reported if there was a task which they were currently supposed to be focused on (Table 1, Question 2). Questions 3–14 ask participants to categorize various qualitative aspects of their mind-wandering state: duration, vividness, enjoyableness, feasibility, tense (past-future), frequency, occupancy, topic, and the person(s) involved, current wakefulness, and mood. Finally, participants were asked to report whether they had consumed any alcohol or drugs (Question 15).

### Procedure

After obtaining informed consent, the mobile phone of the participant was checked to assure its data transfer speed and image presentation ability are compatible with the probe, which they all were. An instruction for the probe session was given to the participants. Participants chose their preferred starting date of 2-week period, and their daily probing schedule from four alternatives: 9:00–21:00, 10:00–22:00, 11:00–23:00, or 12:00–24:00. Once the data sampling started, the probe was sent five times a day for 14 days including weekends. On the last day of the sampling period, an extra probe was sent. Thus, a total number of 71 probes were reached. The probes were sent in pseudo-random time intervals of between 10 and 144 min. The schedule of probing was managed by the Google programming script application GmailDelaySend version 8.0 (Kuzman 2016). The scene closeness rating test and questionnaire were both implemented in Google Forms (Questionnaire accessible at: https://goo.gl/forms/BIJpUNcfbAnwY7aH3).

Participants received a short message for the probe on their mobile device. Each message contains a link to the online forms. Participants tap the link to report their mental state just before the arrival of the notice. They can also decline the probe when necessary, by selecting ‘No, I do not have time now.’ Responses were collected and marked with a time stamp at the Google online forms account.

### Data analysis

All statistical analyses were conducted using the R package (R Core Team 2017) and in-house Python scripts. In order to assess experiential aspects of mind wandering (i.e. relating to possible differences between mind wandering from a task and without a task), responses to the questionnaire items from Table 1 (except for Question 1 asking for the number of thoughts) were analyzed using ANOVA, paired *t*-test and chi-squared tests. This analysis corresponds to Results, Questionnaire summary and Tables 2 and 3.

**Table 2.** Summary of questionnaire results: the current task the mind wandered from

Current task	Mind wandering from a task, Mean and (SD)
School related	53.37 (35.86)
Nothing special	16.86 (26.74)
Social	13.62 (21.41)
Work related	3.90 (9.96)
Commuting	0.93 (2.75)
Others	11.32 (19.73)

**Table 3.** Summary of questionnaire results: experiential aspects of the mind wandering

Questionnaire items	Mind wandering from a task, Mean and (SD)	Mind wandering without task, Mean and (SD)
Duration of mind wandering	10.98 min. (3.62)	10.66 min.(3.29)
5-point ratings		
Vividness	3.39 (0.59)	3.42 (0.85)
Enjoyableness	3.28 (0.68)	3.40 (0.58)
Feasibility	3.94 (0.51)	3.92 (0.45)
Tense (past-future)	3.38 (0.53)	3.44 (0.39)
Frequency	3.00 (0.70)	3.05 (0.62)
Occupancy	3.39 (0.81)	3.52 (0.60)
Wakefulness	3.32 (0.67)	3.26 (0.77)
Mood (sad–happy)	3.37 (0.56)	3.43 (0.55)
Topics of mind wandering (%)		
Yourself	39.82 (31.25)	40.57 (30.01)
Friend(s)	20.46 (21.17)	18.92 (20.68)
Nobody in particular	15.70 (17.51)	15.13 (21.60)
Partner	7.82 (15.22)	13.54 (16.34)
Acquaintance(s)	5.67 (11.18)	3.71 (7.32)
Family member(s)	4.64 (7.15)	6.17 (21.60)
Others	5.88 (12.83)	1.97 (4.77)

Results are shown for mind wandering from a task and mind wandering without task.

**Figure 3.**
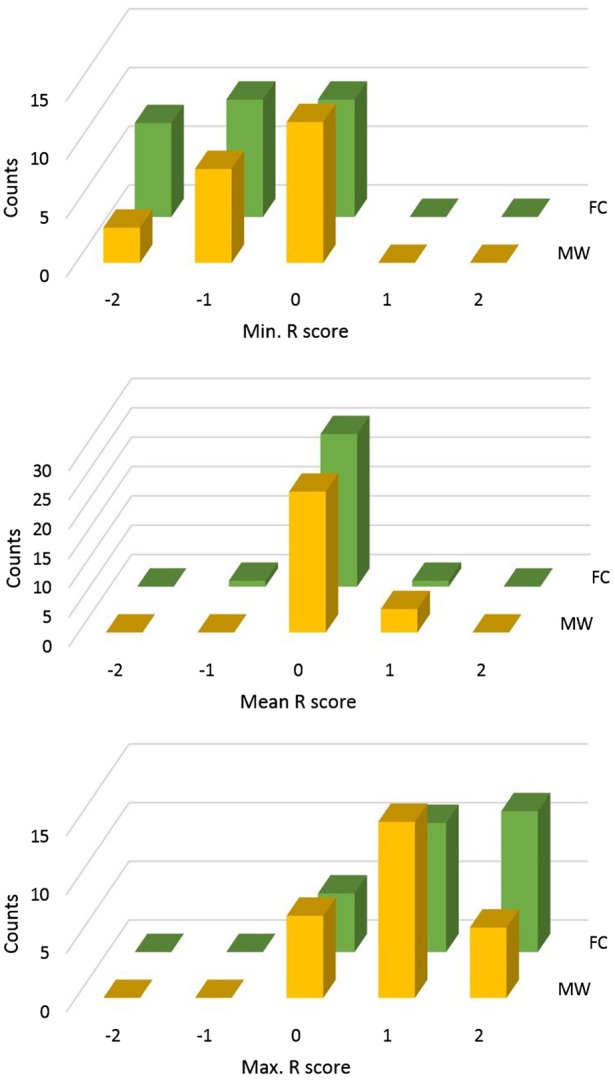
Frequency distributions of individual minimum, mean, and maximum Rating (R) scores. Histograms of the minimum (top), mean (middle), and maximum closeness R scores (bottom). The R scores in mind wandering (MW) and focused (FC) states are shown in yellow and green, respectively.

Prior to trajectory reconstruction, numbers of thoughts (NoTs, Question 1) and scene closeness ratings obtained from the probes were grouped, depending on the answer to Question 2, into task-focused and mind-wandering states. The NoTs in each state were averaged and their difference tested by a paired *t*-test. The scene closeness ratings, “much closer up,” “a little closer up,” “the same,” “a little farther away,” or “much farther away” were scored as −2, −1, 0, 1, and 2, respectively (R scores). Nonzero R scores were taken to indicate top-down bias overruling the veridical “same” response. Mean R scores and other measures of central tendency are mainly determined by the default, unbiased “same” ratings. Hence, they are not the most appropriate statistics for rare and biased ratings. Instead we, used the extreme values (the minimum and the maximum) of R score distributions. Extreme values are commonly used in analyses of rare events (Gomes and Guillou 2015) and dynamical systems analysis (Faranda *et al.* 2013). Certain response strategies could, in principle, interfere with the analysis, such as a tendency to respond that the second image is larger. To control for this possibility, effects observed in the extreme values can only be ascribed to control if they *don’t* occur in the means. Thus, for the R scores, three summary statistics, the minimum, mean, and maximum, were obtained. These, respectively, were compared between focused and mind-wandering states. For this, because extreme value distributions are non-Gaussian and asymmetric, the Brunner–Munzel test (Brunner and Munzel 2000, implemented in R-library “lawstat”) was used. This analysis corresponds to Results, Summary of NoTs and R scores, and Fig. 3.

NoTs and R scores were processed for reconstruction of the mind-wandering trajectories. Here, we outline the processing; details are reported in Supplementary materials, Part 2, Data analysis for trajectory reconstruction. Data were firstly standardized, in order to counteract individual differences in response tendencies: For each participant, we subtracted from the raw R scores their individual minimum, mean, and maximum R score to obtain, respectively: dMin R score, dMean R score, and dMax R scores. For the sake of consistency, the same standardization was applied to the NoTs, yielding dMin NoTs, dMean NoTs, and dMax NoTs. The remaining procedure was applied to each of these six measures.

Next, secondly, the 2 weeks probe time series of each participant was segmented, based on probes in which mind wandering was reported. Each segment extends over a 17-hour period, from 8 hours prior till 9 hours after a report of mind wandering. Since the probe data were obtained at uneven time intervals, in order to convert them to series with equal time intervals, i.e. interval series, probes were binned into 1-hour intervals. This yielded interval series of 17 one-hour binned data (from Bin −8 to Bin 8. Bin 0 included the reference mind wandering) per participant. A grand average over participants was taken for each bin.

Thirdly, probability distributions for the grand averages were obtained through bootstrapping (Efron 1992). Bootstrapped distributions were needed to take into account factors such as uneven numbers of probes across participants and bins, and the temporal dependency of the samples. Each grand average was assigned the percentile value in the corresponding bootstrapped distribution: the higher the percentile NoTs, the higher the number of thoughts (Fig. 5); the higher the percentile R score, the higher the level of deliberate constraints (Fig. 6).

Finally, the trajectories of the mind-wandering process were plotted in terms of percentile NoTs and R scores (Fig. 7).

## Results

### Excluded data

Of the 32 participants, four who responded to less than 40 probes were excluded. Moreover, probes which were not received or responded to properly (e.g. incomplete answers) were discarded. Thus, the remaining analyses were made on 1807 samples from 28 participants. Of the remaining samples, 31 ones, in which participants reported being under the influence of alcohol (Question 15) or experienced display problems in the scene closeness rating test, were excluded from data analysis. Therefore, the total number of valid probes is 1776 (out of the maximally possible 32 × 71 = 2272), on average 61.14 probes per participant [SD = 9.46, 95% confidence interval is (57.48–64.81)].

### Probing period

Of the remaining 28 participants, 6 chose 9:00–21:00, 15 chose 10:00–22:00, and 7 chose 11:00–23:00, as daily probing period.

### Questionnaire results

Data from Question 1 (number of thoughts) were entered to the Interval series analysis to be reported later. This section concerns Questions 2–15. Question 2 asks whether participants experienced mind wandering just before a probe arrived. The participants on grand average reported 62.42% [SD = 17.35; the 95% confidence interval is (55.69–69.14)] of the times that they were focusing on a specific task. Percentage of mind wandering with task was 17.53% [SD = 11.06; the 95% confidence interval is (13.24–21.82)], that of mind wandering without task was 18.28% [SD = 12.43, 95% confidence interval is (13.46–23.09)]. When both types of mind wandering are combined, the percentage (35.81%), is similar to that (32%) reported by Kane *et al.* (2017), in which experience sampling was performed with 274 American undergraduates. Possible influence of the days of week on mind wandering was evaluated by a chi-squared test, but the frequencies did not vary between days of the week, χ^2^ (6) = 2.52, *P* > 0.10, and χ^2^ (6) = 2.77, *P* > 0.10, for mind wandering with and without task, respectively. Thus, for the following analysis the data were collapsed over the days of the week. In case mind wandered from a task, participants reported the types of tasks (Question 12). School-related tasks were reported more than 50% of the times (Table 2).

The duration of mind wandering (Question 3, Table 3) did not differ between with or without task; on average, 10.98 min [SD = 3.62, the 95% confidence interval is (9.57–12.38)] versus 10.66 min [SD = 3.29, the 95% confidence interval is (9.39–11.94)], respectively. A paired *t*-test resulted in *t*(27) = 0.82, *P* > 0.10.

Answers to Questions 4–9, 13, and 14 which involved qualitative aspects of their mind-wandering state are summarized in Table 3. Results of five-point rating questions were compared between mind wandering with and without task using a two-way ANOVA (with/without task by eight questions). The test showed no main effect of task, *F*(1, 423) = 0.59, *P* > 0.10, nor an interaction *F*(7, 432) = 0.15, *P* > 0.10.

On the topics of mind wandering, free form answers (Question 10) varied widely and were sometimes incomplete. As a consequence, we omitted the result from the analysis. The objects of mind wandering (Question 11) were, “yourself” or “friends” in more than 50% of the times; “nobody in particular” and “partner” followed in both types of mind wandering. The percentages for “acquaintance(s)” and “family member(s)” were about 5% and the difference in the rank order between the two types of mind wandering was considered negligible given their standard deviations. These results showed that the topic rankings are practically indistinguishable for the two types of mind wandering. In subsequent analyses, mind-wandering samples with and without a task were pooled.

### Summary of NoTs and R scores

Larger numbers of thoughts were reported in mind wandering than in task focused states, *t*(27) = 9.45, *P* < 0.001. The mean NoTs were 1.86 [SD = 0.65; the 95% confidence interval is (1.00–2.96)] and 1.03 [SD = 0.45; the 95% confidence interval is (0.23–2.07)], respectively.

Frequency distributions of individual minimum, mean, and maximum R scores are shown in Fig. 3 for mind wandering and task focused states, respectively. Note that a score closer to zero (‘the same’ response to the second image) implies a smaller bias, i.e. less deliberate constraints to the task. The distributions, in particular those for the extreme values, were non-Gaussian and asymmetric. To statistically evaluate the difference between two non-Gaussian and asymmetric distributions, the Brunner–Munzel test was used (Brunner and Munzel 2000, implemented in R-library “lawstat”). We report the results in the form: BM (xx) = yy, *P* < zz, where xx represent estimated degrees of freedom, yy the value of the statistic, and zz its significance level. The test results in the minimum values was, BM (53.12) = 2.14, *P* = 0.03. According to this result, deliberate constraints were relaxed during mind wandering compared with focused states. The maximum scores also suggested relaxed deliberate control in mind wandering than in focused states, however, BM (52.45) = −1.53, *P* = 0.13 failed to reach significance. (In a follow-up laboratory experiment both minimum and maximum R score distinguished focused and mind-wandering states. cf. Supplementary materials, Part 1.) According to the means, the two mental states were indistinguishable, BM (50.93) = 0.58, *P* = 0.56. This implies that the effects observed for the extreme values, in particular the minimum, cannot be attributed to specific response tendencies. We may conclude that R scores can distinguish mind wandering and focused states, showing lower deliberate constraints in mind wandering than in task focused states.

### Interval series analysis

#### Summary of binning

Not all bins contain samples from all participants, because of the pseudorandom interprobe interval used (between 10 and 144 min). Bins −8 to +8, respectively, contained one or more samples from 19, 25, 25, 27, 26, 26, 26, 26, 28, 27, 28, 27, 27, 27, 25, 21, and 18 participants (out of max. 28). The number of samples falls off for early and late bins because probes were made over the period of wakefulness, excluding the period between 10:00 PM to 8:59 AM. No segment bridged over successive day times, e.g. *not* Bin −8 at 8:00 PM to 8:59 PM and Bin +8 at 11:00 AM to 11:59 AM of the next day. This means, for instance, that for a mind-wandering report given in response to an 11:00 AM probe, there are no data for the hours between Bins −8 and −3. The difference in number was one of the reasons for using bootstrapping in the data analysis.

**Figure 4.**
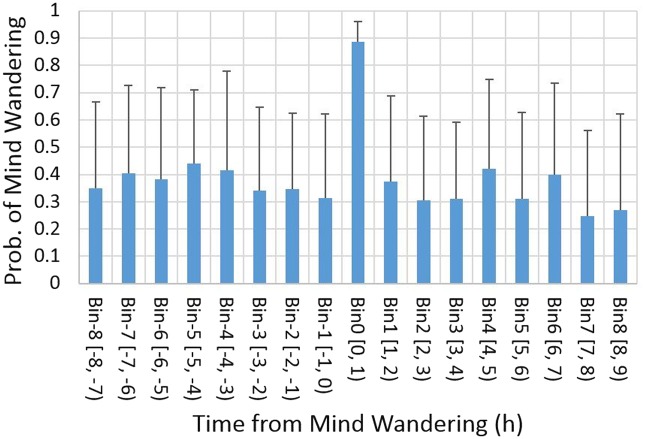
Probability of mind wandering in time. The probability mind wandering is plot for each 1-h time bin. The lower and the upper boundaries of the bins are indicated next to bin numbers along the horizontal axis, e.g. (0, 1) for Bin 0. Error bars indicate SD.

Mind wandering took place more than once within a 12-h probing period, in 10.95% of the 14 days. Because of this, the same data points could appear in different segments. For instance, when mind wandering occurred at 12:00 and 14:30 on a single day, the first segment is cut from the probe at 12:00, and the mind-wandering probes are assigned to Bin 0 and Bin 2. The second segment is cut from the probe at 14:30, and the mind-wandering probes are assigned to Bin 3 and Bin 0. Our results are not distorted by multiple binning of mind-wandering events, as long as they are homogeneously distributed outside of Bin 0. To check for homogeneity, the average probability of a mind-wandering report across participants is computed for each bin. The grand averages are shown in Fig. 4. Note that the probability at Bin 0 does not become one because some reports of focused states were also included in this bin. Excluding Bin 0, a one-way ANOVA (repeated measures, 16 bins) showed no effect of bins on the probability of mind wandering, *F*(15, 384) = 0.76, *P* > 0.10. We conclude that, except for the reference Bin 0, mind-wandering events were homogeneously distributed over the bins.

#### Interval series of NoTs and R scores

Interval series analysis was applied to standardized NoTs and R scores, dMin NoTs, dMean NoTs, dMax NoTs dMin R score, dMean R score, and dMax R score. Given that the minimum R score was shown to be most sensitive to differences in mental states, results in dMean NoTs and dMin R score are shown in the main text. The remaining results are included in Supplementary materials, Part 2.

**Figure 5.**
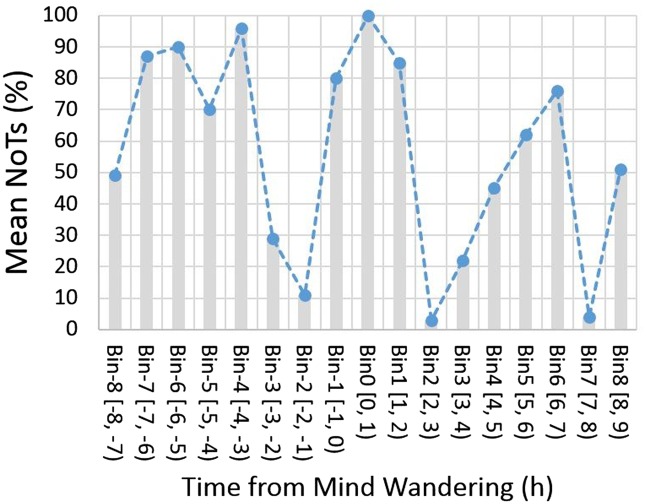
Number of thoughts (NoTs) in time. Time course of NoTs (dMean) is plot in percentile of the corresponding bootstrap distribution. A higher percentile value indicates a higher number of thoughts.

Figure 5 shows the percentile scores of grand average NoTs in time. The graph shows that NoTs oscillates in cycles of 4–6 h. NoTs was in the top percentile at Bin 0. This reconfirms our earlier observation that mind wandering leads to an increase in the number of thoughts concurrently in mind. However, adjacent bins show high scores as well. In Bin −1, i.e. 1 h before the mind-wandering event, the value already reached the 80th percentile and in Bin 1, 1 h after the mind wandering, it was still at the 85th percentile. The high NoTs in the Bins −1 and 1 are not due to the occurrence of mind wandering, which is average in these bins (see Fig. 4). The results clearly showed that high levels of NoTs sustained before and after a mind wandering.

**Figure 6.**
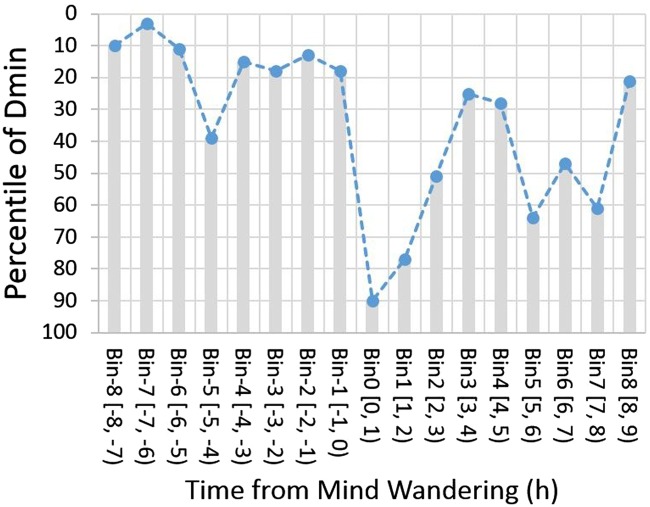
R scores in time. Time course of R scores (dMin) is plot in percentile of the corresponding bootstrap distribution. A higher percentile value indicates stronger cognitive control.

Figure 6 shows the percentile scores of grand average R score in time. The higher percentile values of the score indicate more deliberate constraints. Before mind wandering, the percentile scores were high at relatively constant levels, above the median of the bootstrap data, dropped to the minimum at Bin 0, then slowly recovered from thereon in over the next 4 h.

#### The trajectory of mind wandering

**Figure 7.**
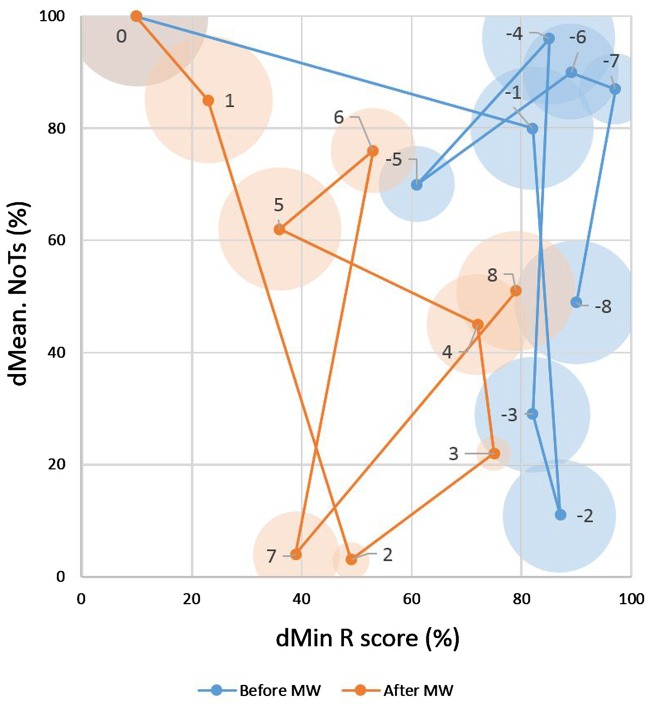
Trajectory of mind wandering. The vertical axis indicates percentile of the number of thoughts (NoTs) of the corresponding bootstrap distribution in dMean. A higher percentile indicates a larger number of thoughts. The horizontal axis indicates the percentile of the scene closeness rating score (R score) of the corresponding bootstrap distribution in dMin. A higher percentile indicates a higher level of deliberate constraints. Number labels next to data points indicate the distance in hourly bins from the mind wandering bin (Bin 0). Blue and orange indicate the trajectory segments before and after mind wandering, respectively. Disk size around each data points indicates inter-individual variability of the trajectory.

Based on the NoTs and the R score, mind-wandering trajectories were reconstructed (Fig. 7). Each point in the trajectory represents a 1-h bin. Disks around the points indicate inter-individual difference in the trajectory (details of the inter-individual difference measure are in Supplementary materials, Part 2). The part of the trajectory before reaching a mind-wandering state is indicated in blue. Eight to 4 h before (Bins −8 to −4), the mind dwells in the upper-right region. This region may be called the “multitasking” region since in this region, the mind is occupied with many thoughts and operates with high deliberate constraints. The trajectory then proceeds for the next 2 h (Bins −3 to −2) to the lower-right region, where the number of thoughts is small and deliberate constraints are high. One hour before the mind wandering (Bin −1) the trajectory rapidly transits back to a state where both the NoTs and the constraints are high. Another rapid transition follows from Bin −1 to Bin 0, the bin where the mind-wandering takes place. The NoTs increase from the 80th to the 100th percentile, while the constraints decrease sharply.

The orange part of the trajectory in Fig. 7 indicates the trajectory after mind wandering. The state 1 h after the interception of mind wandering (Bin 1) shows decrease in number of thoughts, in combination with increase deliberate constraints. The next state (Bin 2) shows continuation of these trends. Between 3 and 8 h after a mind wandering (Bin 3 to Bin 8), the trajectory meanders: the system oscillates and slowly comes back to the median level.

**Figure 8.**
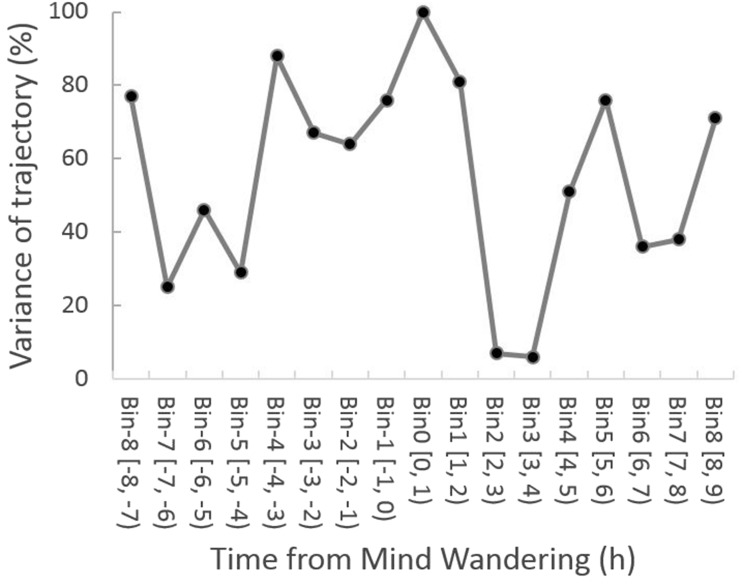
Inter-individual variability in trajectory. Percentile values of the variability in the trajectory are plotted against time bins.

The inter-individual variability in the trajectory is shown in Fig. 8. The trajectory showed a high consistency across individuals for at least some of the time periods. The trajectory converged several hours before mind wandering (Bins −7 and −5), and started diverging afterwards. The divergence peaked at the mind-wandering bin (Bin 0), and then converged again (Bins 2 and 3). The results show that mental states follow a slowly evolving dynamic trajectory in the hours before and after mind wandering.

## General Discussion

Application of experience sampling revealed long-term dynamics of thought generation and cognitive control in the wakeful hours before and after mind wandering. Thought generation rates show a 4–6 h cycle. The cycle falls in the range of ultradian rhythms (1.5–7 h). Ultradian rhythms are a family of biological rhythms observed in behavior, body temperature, cortical EEG, and hormone secretion across mammalian species (Tannenbaum and Martin 1976; Ibuka *et al.* 1977; Ruis *et al.* 1987). Ibuka *et al.* (1977) showed that the ultradian rhythm of 4–7 hours in rats’ behavior and EEG is accentuated by lesion of the suprachiasmatic nucleus, the master circadian rhythm generator. Ultradian rhythms have been related to monoaminergic neuronal populations in the upper brainstem and midbrain that have a key role in arousal promotion (Brown *et al.* 2012). In a mice study of these populations, dopamine neurons had a key role in tuning ultradian rhythms in locomotor behavior (Blum *et al.* 2014). The system for generation and tuning of ultradian rhythms is considered common across mammals. In the current human behavioral study, the 4–6 h cycle of thought generation matches with the daytime activity-rest rhythm, e.g. breakfast at 7:00 AM, course work, lunch at 12:00 PM, course work, tea at 4:00 PM, and so forth. Our results suggest that thought generation could be regulated by ultradian rhythm generators, together with physiological, endocrinological, and behavioral phenomena.

The mind-wandering literature attributes the spontaneous thought generation function to a class of networks, which includes the medial temporal lobule and hippocampus (Blumenfeld *et al.* 2011; Ellamil *et al.* 2012; Beaty *et al.* 2015; Christoff *et al.* 2016). It is an open question, how this functional network is affected by the ultradian rhythm. A recent study on the emotion network (which includes amygdala, insula, striatum, and orbitofrontal cortex) suggests that the endocrinological ultradian rhythm, in particular that of the plasma glucocorticoid level, keeps this network tuned to emotional stimuli (Kalafatakis *et al.* 2018). Subsystems of the thought generation network, such as hippocampus, are also sensitive to glucocorticoids. Thus, the ultradian rhythm might also regulate the responsivity of the thought generation system.

The dimension of cognitive control showed a pattern, different from the 4–6 h cycle of the number of thoughts. Deliberate constraints are sustained at high level before mind wandering, until a rapid drop occurs around 1 h before mind-wandering onset. Afterwards, the trajectory bounces back but eventually settles on moderate values of deliberate constraints. This pattern might be explained in terms of self-feedback of the cognitive control system. As the level of control starts falling, self-feedback pushes the level up. The “bounce back” suggests a delay, or integral gain, of the feedback. Functional MRI studies map cognitive control function to coactivation of anterior cingulate cortex/presupplementary motor area, dorsolateral prefrontal cortex, inferior frontal junction, anterior insular cortex, dorsal premotor cortex, and posterior parietal cortex (Duncan and Owen 2000; Schneider and Chein 2003; Brass *et al.* 2005; Chein and Schneider 2005). The studies did not discuss BOLD activity of the network in terms of self-feedback. However, some of the results are suggestive. For example, BOLD activity of the network alternated “push” (one and more BOLD peaks) and “relaxation” (low or no peak) periods in the time scale of minutes (Cole and Schneider 2007). Our observations may suggest that neural mechanisms of cognitive control may vary dynamically on the time scale of hours as well.

Whereas both the number of thoughts and the deliberate constraints measure suggest long-term regularity, their relationship as shown on the trajectory is far from a simple correlation. Seven to 5 h before mind wandering, participants were within the “multitasking” state with relatively small interparticipant variability. Around 4 h before mind wandering, the interparticipant variability increased, suggesting the participants were escaping at different rates from “multitasking” to a state more amenable to single-task focusing. About 1 h before the mind wandering, participants moved back to a “multitasking” state. Then, the level of deliberate constraints quickly decreases. As, thus, cognitive control was suddenly lost, participants drifted into mind wandering. Afterwards, the participants moved to a state with a small number of thoughts. A segment 2–3 h after the mind wandering showed low interparticipant variability. The probe made the participants aware of their mind wandering. The induced awareness could let cognitive control increase the level of constraints more or less in the same timing for all participants.

Such intricate coordination between the thought generation and cognitive control systems could be implemented via activity of locus coeruleus norepinephrine system (LC-NE) (Mittner *et al.* 2016). LC-NE receives input from the cortical cognitive control network and the subcortical biological rhythm generators, and projects throughout the brain. It shows slow (tonic) activity and fast (phasic) activity which are related to attention control, such as switching of attention to incoming sensory information. The interaction of these subnetworks determines the state of the whole central nervous system. The coordination observed in the dynamics of mind wandering may be a product of this interaction.

The relationship between metacognitive awareness of mind wandering and cognitive control is listed as one of the open questions in the phenomenology of mind wandering (Smallwood and Schooler 2006). The prompts sent during experience sampling might, in principle, elicit a level of metacognitive awareness that disrupts the mind wandering. This may explain the consistency, with which participants afterwards move to a state with a small number of thoughts. On the other hand, in real life, disruptions are the order of the day, so the probes may not retain their salience long enough have an exceptional impact on the mind-wandering process. The unfinished business resulting from the disruption (Zeigarnik 1938) may, in principle, have impelled the continuation of mind wandering after the probe. Therefore, how natural and induced metacognitive awareness influence to cognitive control remains as one of the open questions.

Vice versa, the slow dynamics inevitably affects to events on the faster time scale. For example, lapses of attention (Cheyne *et al.* 2006) and intentional control over mind wandering (Seli *et al.* 2016) have been observed and described in a time scale faster than that in the current study. We would expect to find that these processes are modulated by the long-term trajectories observed in our 2D state space.

From the trajectories we could, in principle, isolate a classical mind-wandering cycle: loss of control, proliferation of thoughts, mind wandering, resumption of control, decrease of thought proliferation. However, whereas such a description is based around discrete events in the state space, the trajectories are continuous. Order of events does not equal causality; this sequence is embedded in a long-term regularity, spanning several hours before and after a mind-wandering event. Moreover, the discrete events are not necessarily in the focal regions where individual trajectories converge, like the uniform movement 2–3 h after mind wandering. Future research may benefit from the identification of possible underlying variables, which we were able to perform with a simple observational method; in particular, the identification of long-term context as relevant for the incidence of mind wandering.

## Supplementary Material

niz007_Supplementary_Data
