# Supplementary material for: Long-term dynamics of mind wandering: ultradian rhythms in thought generation
Source: Neurosci Conscious. 2019 Jun 8;2019(1):niz007. doi: 10.1093/nc/niz007 (PMC6555903; doi:10.1093/nc/niz007)
Supplement: niz007_Supplementary_Data [file niz007_supplementary_data.docx]

**Supplementary materials**

to ‘Long-term dynamics of mind wandering: ultradian rhythms in thought generation’

by Chie Nakatani, Benjamin Ganschow, & Cees van Leeuwen

**Part 1: A reliability study for scene closeness rating test**

The scene closeness rating test was specifically developed for this study. The main statistics of the test are extreme values (the minima and the maxima) of R score distributions. The minima took lower values in self-reported mind wandering than in task-focused mental states. The same pattern was observed numerically in the maxima; however, it was not statistically significant. (see Results, Summary of NoTs and R scores (in the main text). This might be a consequence of noise variations in the conditions of measurement, but may also raise doubts on the reliability of the test. To establish whether extreme values could reliably be used to distinguish focused and mind wandering states, we replicated (emulated?) the scene closeness rating test in experimental conditions.

**Methods**

*Participants*

Twenty healthy volunteers (14 females, mean age = 22.9 years old, SD = 2.9 years).

*Stimuli and display sequence*

Stimuli were the 71 scene images from the scene closeness rating task used in our real-life sampling procedure. Each image was pasted in a frame which resembled that of a smartphone. Image size was 700x700 pixels. The fixation/mask pattern was also presented in the simulated smart phone frame. The simulated smartphone display was presented on a 17-inch computer monitor against a grey background (Figure S1-1). Presentation durations were identical to those used in the real-life sampling (Figure 2 in the main text). The five point-rating screen was presented after the stimulus presentation. Participants chose one of ‘farther away’, ‘little farther away’, ‘same’, ‘little closer’, or ‘closer’.

=== Figure S1-1 ==


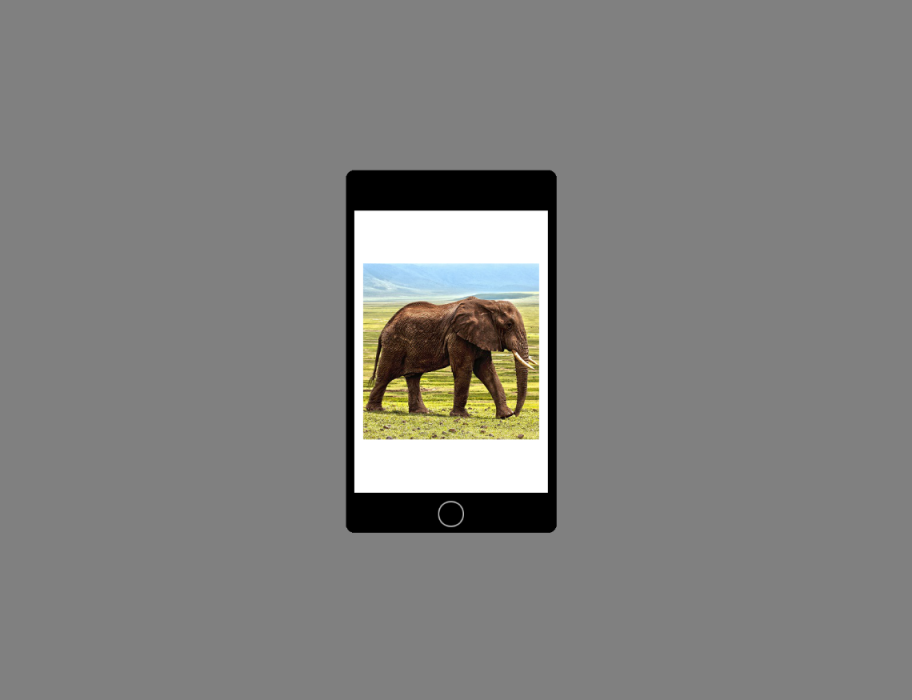


**Figure S1-1**. Example of stimulus display

Stimuli were presented in the simulated smartphone frame on a computer display (gray background).

*Tone counting task*

To induce mind wandering during the experiment, a tone counting task was applied. A simple tone (pitch: 440Hz, duration: 100ms) was repeatedly presented. SOA was varied between 2.0 to 2.5s pseudo randomly. Participants counted the tones up to a target number between 20 and 24, which was chosen pseudo randomly. The target number was presented at the beginning of each trial. When the count reached to the number, participants pressed the ‘5’ key on a ten-key box. Immediately after the keypress, they were asked to score their level of mind wandering during the tone counting task between 0 and 100% in steps of 10 %, using the ten keys of the keyboard.

*Procedure*

Participants were seated in front of the display. Viewing distance was approx.120cm, and viewing angle of the simulated smartphone frame was approx. 6.5 by 8 degrees. The angles were chosen to roughly match those of an actual 5.1- 6.2 inch hand-held smartphone display at a distance of 50cm. Ambient lighting was dim and constant for each participant.

A trial started with the presentation of a target number for the tone counting task. Participants pressed an arbitrary key to start tone presentations. Once the number of the tones reaches the target number, participant pressed the ‘5’ key and reported the percentage of the mind wandering using the ten keys. A scene closeness rating followed immediately. For each trial, one image was chosen from the 71 images pseudo randomly without repetition. The participant reported ‘farther away’, ‘little farther away’, ‘same’, ‘little closer’, or ‘closer’ pressing a corresponding key on the ten-key pad. After the rating, participants retrospectively reported the number of thoughts (NoTs) during the tone counting task. A trial (counting task, scene closeness rating, and NoTs report) together took approx. 1 min. Total 37 trials were completed per participant. The experiment was controlled by a Python script on PsychoPy 1.82.01.

**Results**

Performance of the tone counting task was 72.12% correct on average (SD= 24.15). Given that mind wandering is the focus of the study, error trials were included in the following analysis. Scene closeness ratings , ‘farther away’, ‘little farther away’, ‘same’, ‘little closer’ and ‘closer’ were scored to -2, -1, 0, 1, and 2, respectively. All statistical analyses were performed using the R package (R Core Team, 2017).

A histogram of the reported percentage of mind wandering showed two peaks, one at 35% and the other at 85% (Figure S1-2). The minima between the two peaks (70%) was used to split the trials into low and high mind wandering trials. Four participants had no high mind wandering trials. Their data was excluded from the following analysis.

=== Figure S1-2 ==


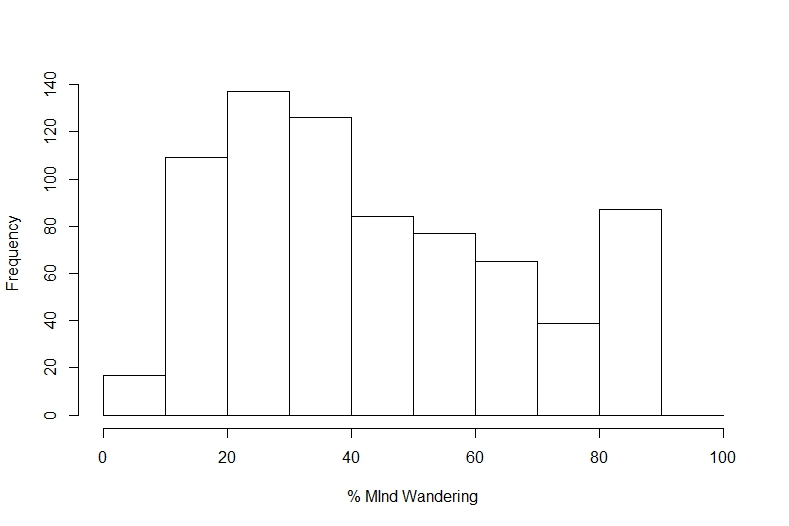


**Figure S1-2.** Frequency distribution of reported mind wandering (in percentages) during the counting task

In each participant, mean rating scores were computed for the low and high mind wandering trials. Grand means were 0.191 and 0.211 for the low and high mind wandering trials, respectively. A paired t-test showed no difference between the grand means, t (15) = 0.10, p = 0.92, consistently with our observation in real life sampling.

Next, the minimum rating score was taken in each participant for the low and high mind wandering trials. The Brunner-Munzel test applied to the minimum score distributions yielded BM (33.71) = -4.43, p < 0.0001. I.e., the minimum score distinguishes low and high mind wandering states. The result replicates that in the real life sampling in the current study. In the maximum rating scores, the Brunner-Munzel test yielded BM (29.28) = 3.15, p = 0.004. The maximum score distinguished the low and high mind wandering states in the laboratory better than in real life sampling.

**Conclusion**

The extreme value statistics (minima and maxima) of the rating scores reliably distinguish mind wandering and task focused states in laboratory conditions. As for the minima, this result replicates that of real-life sampling versions of the task. The maxima distinguished these states better in the laboratory than in real-life sampling. The difference could be attributed, at least in part, to reduced control over task administration in the smartphone version of the test.

**Part 2: Data analysis for trajectory reconstruction**

**Standardization**

Normalization (z-scoring) is not appropriate for statistics based on extreme values, as this would involve adjusting the score according to the mean. Therefore, to the R scores (and for the sake of consistency also to the NoTs), instead we applied standardization to counteract individual differences in response tendencies. For every $X_{i}$, which is a score (NoTs or R) from Probe $i$ out of a total of $m$ probes, we computed: ${dMin=X}_{i}-X_{min}$, ${dMean=X}_{i}-X_{mean}$, and ${dMax= X}_{i}-X_{max}$ , $1\leq i\leq m$, in each participant. This resulted in six standardized measures: dMin R score, dMean R score, dMax R score, dMin NoTs, dMean NoTs, and dMax NoTs (Fig. S2-1). The rest of the procedure was applied to each of these measures.


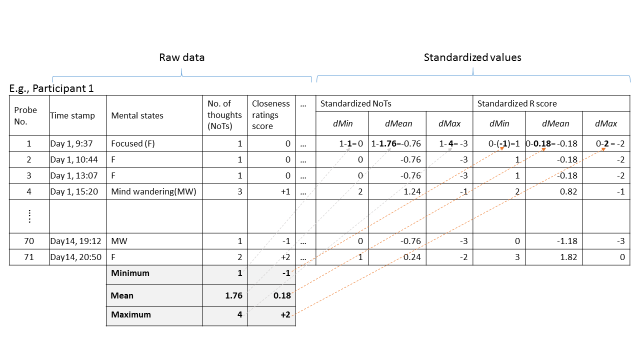


**Fig. S2-1. Standardization of NoTs and R scores**

**Segmentation and binning**

In each participant, the two weeks’ time series of probes was segmented, using mind wandering reports as reference points. The time stamp of each probe in which mind wandering was reported was treated as the zero time point (Time0) of a time segment, starting from 8 hours prior (-8) till 9 hours afterward (+9). Because mind wandering could occur more than once within an 9 hours’ time span, overlapping segments could occur (Fig. S2-2). All segments were aligned from Time0 and aggregated within participants as follows: Across segments, samples are assigned to 1-hour bins. Samples from Time0 to 59 min. are assigned to Bin0. As a result, this bin contains mostly mind wandering samples, but any ‘focused’ responses to probes occurring within the same 1-hour time span are also included. Samples from 60 to 119 min. after Time0 are put to Bin1 and samples from 60 to 0 min. before Time0 are put to Bin-1. Likewise, samples between -8 and 9 hours from mind wandering were assigned to bin n, with n ϵ {- 8, …, +8}, yielding a total of 17 bins. For each of our standardized measures, bin averages were computed per participant. Subsequent bin averages constitute interval series. (Fig. S2-3).


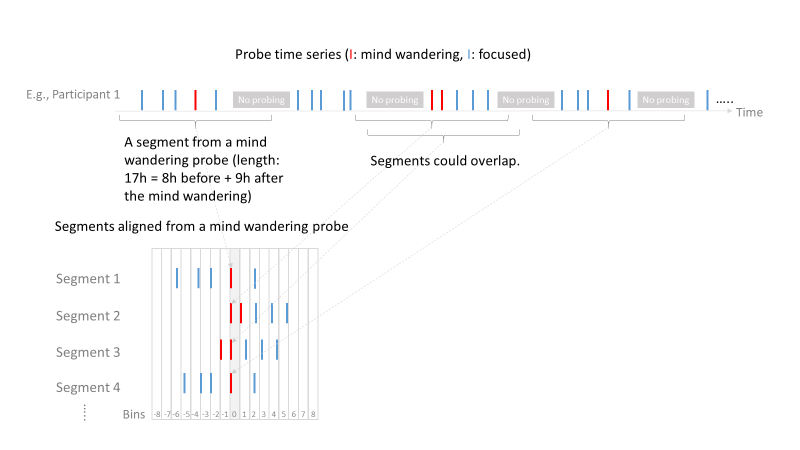


**Fig. S2-2.** Segmentation and binning of probe time series


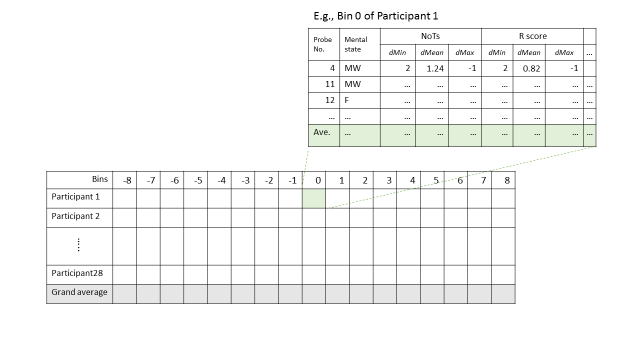


**Fig. S2-3.** Data structure of interval series

**Bootstrapping**

Grand averages in each bin were computed over participants. Next, the grand averages were scaled to percentile values in reference to a bootstrap distribution (Efron, 1992) of grand averages, which was obtained as follows. First, for each participant, a number *n* of arbitrary probe responses were chosen randomly from his or hers two-weeks sampling period, with *n* being the same number as the number of probes in which the participant reported mind-wandering. These randomly selected probes were assigned a pseudo timestamp Time0. Pseudo-segments between -8 and +9 hours were taken to generate surrogate bin data (Fig. S2-4). From the surrogate data of all participants, surrogate grand averages were computed for each bin. The procedure was repeated for 1000 times to generate distributions of 1000 surrogate grand averages per bin. These distributions share with the real data all characteristics of different numbers of samples across bins and participants and possible temporal dependencies of the measures. The real grand averages were assigned the percentile scores according their vales in the surrogate distributions (Fig. S2-5). For example, the percentile of the grand average of dMean NoTs at Bin0 being 97% means that the real bin grand average corresponds to the 3% highest values in the grand average dMean NoTs surrogate distribution.


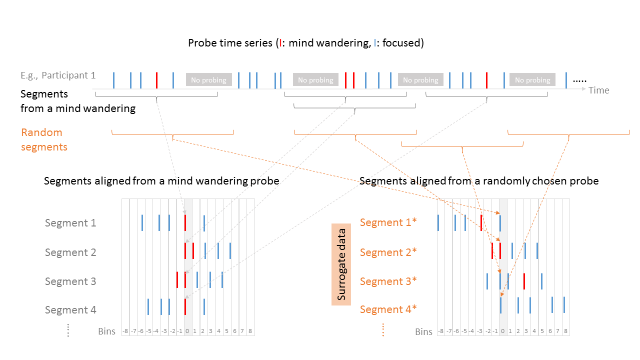


**Fig. S2-4**. Illustration of surrogate data generation


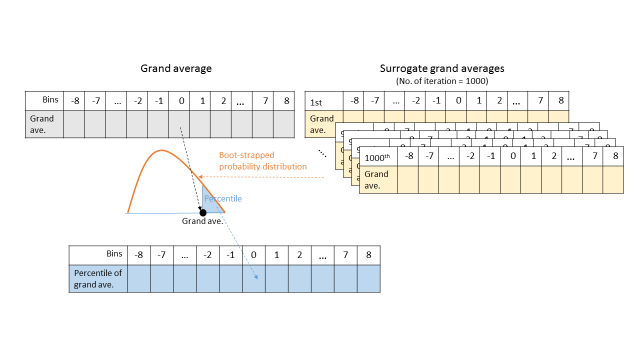


**Fig. S2-5.** Percentile of grand averages

**Results of interval series analysis in six measures**

The analysis involving segmentation and bootstrapping was applied to all six measures: dMin NoTs, dMean NoTs, dMax NoTs, dMin R score, dMean R score, and dMax R score. Of the six sets of results, the main text only shows those of dMean NoTs and dMin R scores. As supplementary information, we present all six results. Fig S2-6 shows the results in dMin, dMean, and dMax NoTs. A higher percentile value indicates a higher number of thoughts. The results were similar among the three measures. Likewise, Fig S2-7 shows the results in dMin, dMean, and dMax R scores. A higher percentile value indicates stronger deliberate control. Again, the results were similar among the three measures, with a possible exception involving Bin+8.

**Fig. S2-6.** Time course of NoTs computed from dMin, dMean and dMax measures

**Fig. S2-7.** Time course of R scores computed from dMin, dMean and dMax measures

**Trajectory of mind wandering**

A trajectory of mind wandering was drawn combining the percentile values of dMean NoTs and dMin R scores (Figure 7 in the main text). For each time bin, a measure of inter-individual variability was computed as follows: From the grand average, $\bar{a}$ , computed over individual dMean NoTs, $a_{i}, 1\leq i\leq28$ and grand average, $\bar{b}$, computed likewise over individual dMin R scores, $b_{i}$, the Euclidian distance between the grand average and the individual points:

$${Distance}_{i}= \sqrt{\left( a_{i}-\bar{a} \right)^{2}+\left( b_{i}-\bar{b} \right)^{2}}$$

was computed and averaged over participants. To evaluate the variability, 1000 pseudo distance averages were computed from the 1000 surrogate data from the same bin and a percentile value was assigned to the real average distance corresponding to that in the surrogate averages distribution. These percentile values constitute our variability measure. A high percentile value indicates that the inter-individual variability at the bin was large. The percentile values of all time bins were shown in Figures 7 and 8 in the main text.
